# Joint Cardiac *T*_1_ Mapping and Cardiac Cine Using Manifold Modeling

**DOI:** 10.3390/bioengineering10030345

**Published:** 2023-03-09

**Authors:** Qing Zou, Sarv Priya, Prashant Nagpal, Mathews Jacob

**Affiliations:** 1 Division of Pediatric Cardiology, Department of Pediatrics, The University of Texas Southwestern Medical Center, Dallas, TX 75390, USA; 2 Advanced Imaging Research Center, The University of Texas Southwestern Medical Center, Dallas, TX 75390, USA; 3 Department of Radiology, The University of Texas Southwestern Medical Center, Dallas, TX 75390, USA; 4 Department of Radiology, The University of Iowa, Iowa City, IA 52242, USA; 5 Department of Radiology, University of Wisconsin–Madison, Madison, WI 53792, USA; 6 Department of Electrical and Computer Engineering, The University of Iowa, Iowa City, IA 52242, USA

**Keywords:** variational autoencoder, generative model, CNN, manifold approach, unsupervised learning, cardiac MRI, image reconstruction

## Abstract

The main focus of this work is to introduce a single free-breathing and ungated imaging protocol to jointly estimate cardiac function and myocardial $T_{1}$ maps. We reconstruct a time series of images corresponding to k-space data from a free-breathing and ungated inversion recovery gradient echo sequence using a manifold algorithm. We model each image in the time series as a non-linear function of three variables: cardiac and respiratory phases and inversion time. The non-linear function is realized using a convolutional neural networks (CNN) generator, while the CNN parameters, as well as the phase information, are estimated from the measured k-t space data. We use a dense conditional auto-encoder to estimate the cardiac and respiratory phases from the central multi-channel k-space samples acquired at each frame. The latent vectors of the auto-encoder are constrained to be bandlimited functions with appropriate frequency bands, which enables the disentanglement of the latent vectors into cardiac and respiratory phases, even when the data are acquired with intermittent inversion pulses. Once the phases are estimated, we pose the image recovery as the learning of the parameters of the CNN generator from the measured k-t space data. The learned CNN generator is used to generate synthetic data on demand by feeding it with appropriate latent vectors. The proposed approach capitalizes on the synergies between cine MRI and $T_{1}$ mapping to reduce the scan time and improve patient comfort. The framework also enables the generation of synthetic breath-held cine movies with different inversion contrasts, which improves the visualization of the myocardium. In addition, the approach also enables the estimation of the $T_{1}$ maps with specific phases, which is challenging with breath-held approaches.

## 1. Introduction

Magnetic resonance imaging (MRI) is commonly used for the diagnosis and prognosis of cardiac disorders. The improved image contrast, clarity, and non-iodizing nature of image acquisition make cardiac MRI the gold standard for cardiac function and tissue characterization. Cardiac function is usually measured using gated breath-held 2D acquisitions [1,2]. The changes in myocardial tissue composition often result in variations in the $T_{1}$ relaxation time, which can be measured using $T_{1}$ mapping sequences; these approaches have shown great potential in the detection of myocardial pathologies and therapy monitoring [3,4,5]. Commonly used $T_{1}$ sequences include breath-held modified Look-Locker inversion recovery (MOLLI) [6], saturation recovery single-shot acquisition (SASHA) [7], and saturation pulse prepared heart rate independent inversion recovery (SAPPHIRE) [8]. The cardiac function and $T_{1}$ mapping acquisitions often require one breath-hold per slice and intermittent rest periods between the acquisition of the slices. The acquisition of the above datasets from multiple slices to cover the whole heart translates to long scan times, in addition to reduced patient comfort.

Compressed sensing and low-rank methods have been extensively used to reduce the breath-hold duration in cardiac cine [9,10] and $T_{1}$ mapping [11,12]. MR fingerprinting methods have been introduced for $T_{1}$ and $T_{2}$ mapping of the myocardium [13,14]. These schemes rely on dictionaries of magnetization evolution, obtained using Bloch simulation. All of these approaches require electrocardiogram (ECG) gating and breath-holding, which is not tolerated by several subject groups, including pediatric subjects. Several elegant approaches exploit the synergies between cine MRI and $T_{1}$ mapping. For instance, refs. [13,15] use the central k-space samples in breath-held inversion recovery sequences to determine the cardiac phases, followed by the recovery of the magnetization evolution of the signals from each phase using low-rank and spatial and temporal regularization. The temporal subspace basis functions were derived using Bloch simulations in both cases [13,15]. Recently, free-breathing approaches were introduced to extend the approach to patient populations that cannot hold their breath. For instance, MR multi-tasking [16] relies on a low-rank tensor model for the multi-dimensional signal to relax the breath-holding requirement. This approach estimates the tensor factors from dedicated k-space navigators, which are acquired at every other readout. The constrained tensor model quite significantly reduces the data demand, making the motion-resolved recovery of the multi-dimensional dataset from highly undersampled measurements well-posed. The need for dedicated navigators makes it challenging to adapt this scheme to general sequences. In addition, the use of continuous acquisition may make it difficult to decouple the effects of flip angle and $T_{1}$ recovery, which may affect the accuracy of the estimated $T_{1}$ maps. To overcome this issue, Zhou et al. [17] introduced a dual flip angle approach, where the acquisition consists of two blocks of two different flip angles. They perform a low-rank and sparse joint recovery of cardiac phase and $T_{1}$ dynamics that is conceptually similar to [13,15], while compensating for translational respiratory motion.

The main focus of this work is to introduce a free-breathing and ungated imaging protocol for the joint acquisition of cardiac function and $T_{1}$ maps. We capitalize on the synergies between functional imaging and $T_{1}$ mapping to reduce the scan time and to improve patient comfort. We recover the images with different contrasts from an inversion recovery gradient echo (GRE) sequence using a generative deep learning model, which is learned in an unsupervised fashion. We consider each image in the time series as a non-linear function of the cardiac and respiratory phases as well as the inversion time. Similar to our prior work [18], we model the non-linear function by a convolutional neural network (CNN). The parameters of the CNN, as well as the latent variables corresponding to the cardiac and respiratory phases, are learned from the measured undersampled k-t space data of the same patient, resulting in a subject-specific representation. Because the inversion time is known as a priori, we use it as a preset latent vector that is not learned during the optimization process. When the latent variables vary in a 3D Cartesian space, the corresponding images vary in a smooth 3D surface or manifold in a high-dimensional space. Hence, this approach essentially uses a manifold constraint on the images. The non-linear representation allows us to represent the data using a few parameters that are directly related to the physical parameters (phase information and inversion time). The G-SToRM formulation was originally introduced for motion-resolved cardiac cine MRI; the images in the dataset are modeled as non-linear functions of low-dimensional latent variables that represent the cardiac and respiratory phases. We use a coarse-to-fine progressive CNN architecture, which allows us to represent the global features, including the shape and contrast of each image frame in the low-resolution initial layers. The high-resolution layers add finer details may be the same for all frames. We use a continuous-acquisition strategy using a gradient echo (GRE) spiral acquisition scheme with intermittent inversion pulses and delays for magnetization recovery, while the data were acquired in a free-breathing and ungated mode.

We introduce an efficient two-step reconstruction algorithm, which is significantly more computationally efficient than our previous joint estimation strategy [18,19]. We use a variational auto-encoder (VAE) for the estimation of the latent vectors from the central k-space samples. Once the latent vectors are estimated, the CNN model is trained with the latent vectors fixed. Once the learning of the CNN is complete, we can excite the CNN with arbitrary latent vector combinations to obtain $T_{1}$ weighted images with arbitrary cardiac and respiratory phases. We estimate the $T_{1}$ maps by Bloch equation mapping.

We note that generative modeling is emerging as a powerful tool in medical imaging [20,21]. We have also introduced motion-resolved (SToRM) [18] and motion compensated (MoCo-SToRM) [22] approaches for dynamic MRI applications. The G-SToRM manifold model can be viewed as a non-linear generalization of low-rank [11,13,15] and tensor models [16], where the images are modeled as linear functions of the temporal basis vectors (e.g., cardiac, respiratory motion vectors). Note that low-rank and tensor models learn the basis vectors that are non-linear functions of the physical parameters from k-t space navigators.

## 2. Background

### 2.1. Dynamic MRI Recovery

The main objective in dynamic MRI is to acquire high-resolution images from possibly undersampled measurements. The image frames $x_{i}:i=1,\cdots ,M$ in the time series are often compactly represented by the Casoratti matrix:

(1) $$X=\left[\begin{array}{ccc} x_{1} & \cdots & x_{M} \end{array}\right].$$

The MR images are acquired by multichannel measurement operators, which are often different for each image:

(2) $$b_{i}=A_{i}\left(x_{i}\right)+n_{i}.$$

Here, $n_{i}$ is a zero-mean Gaussian noise matrix that corrupts the measurements. Specifically, $A_{i}$ are the time-dependent measurement operators, which evaluate the multi-channel Fourier measurements of the image frame $x_{i}$ on the trajectory $k_{i}$ corresponding to the time point *i*.

### 2.2. Single-Step Cardiac Cine MRI Using Generative SToRM

CNN-based generative models were recently introduced for free-breathing and ungated cardiac MRI [18,19] without any contrast variations. This scheme models the 2D images in the time series as the output of a CNN generator $G_{\theta }$:

$$x_{i}=G_{\theta }\left(z_{i}\right),\;i=1,\cdots ,M.$$

The input $z_{i}$ is the latent vector, which lives in a low-dimensional subspace. The recovery of the images in the time-series involves the minimization of the criterion

(3) $$\begin{array}{cc} C(z,\theta )= & \underset{\mathrm{data}\;\mathrm{term}}{\underset{︸}{\sum_{i=1}^{M}{∥A_{i}\left(G_{\theta }\left(z_{i}\right)\right)-b_{i}∥}^{2}}}\\ \; & +\lambda_{1}\underset{\mathrm{net}\;\mathrm{reg}.}{\underset{︸}{∥J_{c}G_{\theta }{\left(z\right)∥}^{2}}}+\lambda_{2}\underset{\mathrm{latent}\;\mathrm{reg}.}{\underset{︸}{∥\nabla_{i}z_{i}{∥}^{2}}}. \end{array}$$

The first term in the cost function is a measure of data consistency. The second term penalizes the norm of the Jacobian of the deep network, which serves as a regularization term; it controls the smoothness of the generated image manifold [18]. The last term penalizes the temporal gradient of the latent variables, which facilitates the recovery of smooth latent vectors that are free of rapid changes that may arise from alias artifacts. Instead of reconstructing the images, the G-SToRM approach learns the optimal latent vectors and the CNN parameters from the measured data. Once the learning is complete, we generate the images in the time-series by exciting the generator with appropriate latent vectors, often corresponding to the respective cardiac/respiratory phase.

## 3. Methods

### 3.1. Acquisition Scheme

**Figure 1:**
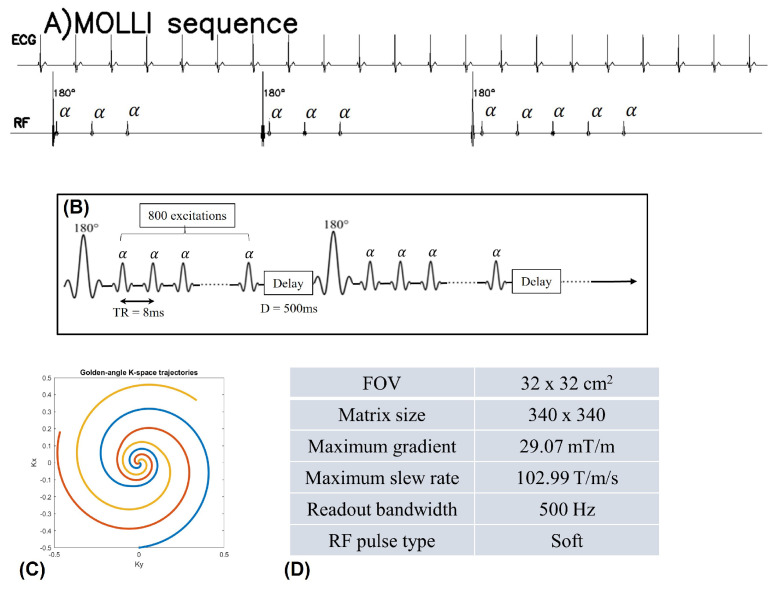
(**A**) shows the MOLLI sequence scheme. (**B**–**D**) show the inversion recovery sequence for free-breathing and ungated cardiac MRI. The acquisition is started with a 180${}^{\circ }$ inversion pulse at the beginning, and following inversion pulses were applied every 6.9 s (6.4 s of data acquisition and 0.5 s of delay time). The pulse diagram is shown in (**B**). The data are continuously acquired with golden angle (137.5${}^{\circ }$) spiral trajectories (**C**). In (**D**), we list the detailed sequence parameters. ((**A**) is adopted from Figure 1 in [23] with permission).

The proposed sequence for the data acquisition in this work is depicted in Figure 1. The free-breathing and ungated cardiac data are acquired using a continuous spiral sampling of the k-space with golden angle increment and GRE readouts. The longer repetition time (TR) offers enhanced inflow contrast between the myocardium and blood pool compared to the shorter TR Cartesian GRE sequences. In addition, the spoiled GRE sequence for the acquisition does not suffer from banding artifacts, unlike steady-state free precession (SSFP) sequences. In addition, the GRE sequence with a spiral acquisition scheme is much less sensitive to the eddy-current effects, and hence we do not need to correct the trajectories before reconstruction. We use a constant flip angle for the GRE readouts. We use inversion blocks of duration ≈7 seconds, consisting of an adiabatic inversion pulse, 800 spiral interleaves with TR =8 ms, and a delay of 500 ms for longitudinal magnetization relaxation. The readout duration of the spirals is 2.74 ms. Detailed parameters for the sequence used in this work can be found in Figure 1.

### 3.2. Pre-Estimation of the Latent Variables Using VAE

In our previous work [18,19], the CNN parameters as well as the latent vectors were jointly estimated from the entire k-t space data. A challenge with this approach is the high computational complexity. In addition, this approach is not directly applicable to the $T_{1}$ mapping setting considered in this work because the navigators are modulated by the inversion pulses.

**Figure 2:**
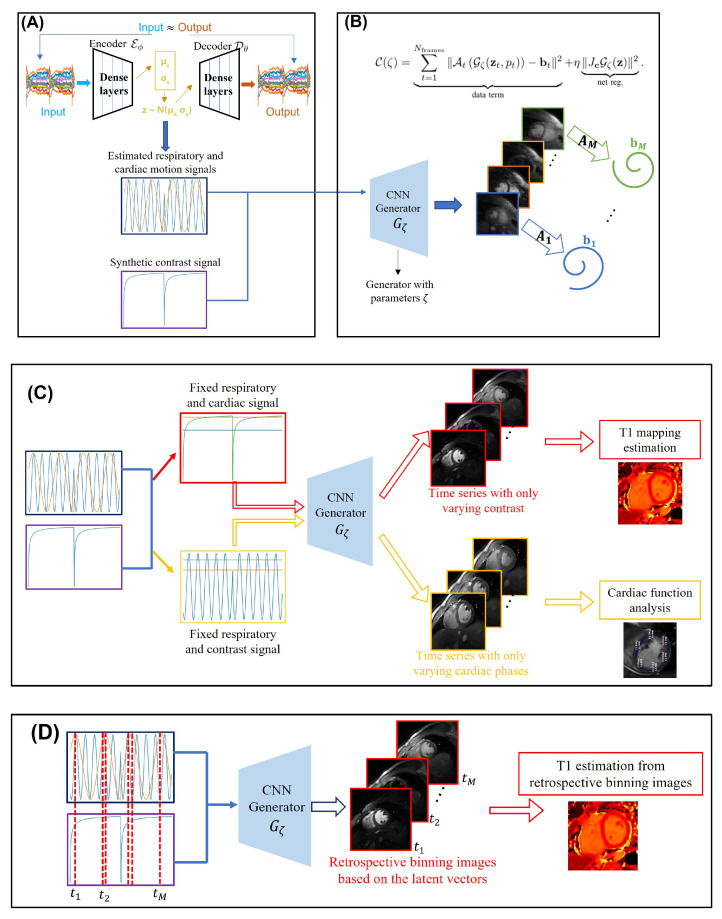
Illustration of the proposed two-step reconstruction scheme. (**A**) shows the estimation of the latent signals from the k-t space navigators using a variational auto-encoder. The center k-space data are used as the input of the encoder, and it tries to learn the motion distribution. (**B**) depicts the reconstruction framework. The estimated latent signals together with the contrast signal, derived from the timing of the inversion pulses, are fed into the generator with parameters θ. The generator then outputs the image frames in the time series with varying contrast, cardiac phase, and respiratory phase. The forward operators are applied for each image frame and compared to the acquired k-space measurements. Post-recovery, the framework can be used in a generative mode as shown in (**C**). For instance, we fix the respiratory and cardiac signals and only vary the contrast signal. These signals are then fed into the learned generator, which then outputs the image frames in the time series that correspond to different inversion times, which are then used for the $T_{1}$ estimation. Likewise, the respiratory and contrast signals can be fixed and the cardiac signal can be varied; we can obtain a synthetic breath-hold cine by feeding this combination to the generator. The retrospective binning strategy for $T_{1}$ mapping estimation is illustrated in (**D**). Here, we identify the time-instants when the latent vectors are in a specified cardiac and respiratory phase, and we feed the generator the corresponding latent vectors and inversion signal to generate the corresponding images. The $T_{1}$ maps are obtained by matching the images with the sub-dictionary of the fingerprints.

To minimize the computational complexity and to adapt the method to the $T_{1}$ mapping setting, we propose a two-step strategy illustrated in Figure 2A,B; in the first step, we estimate the cardiac and respiratory phases from the k-space navigators (central k-t space data) from all the coils using a conditional VAE [24] as shown in Figure 2A. We assume the latent variables $c_{t}$ to be two-dimensional, with the dimensions corresponding to the cardiac phase and respiratory phase, respectively. Because the timing of the inversion pulses are known a priori, we feed the inversion signal $p_{t}$ as a conditional vector to the network, which is held fixed throughout the optimization process. In this work, we use a three-layer fully connected network with three inputs, obtained by the concatenation of $c_{t}$ and $p_{t}$.

The VAE consists of an encoder $E_{\phi }$ and a decoder $D_{\theta }$

(4) $$\begin{array}{ccc} \left(z_{t},\sigma_{t}\right) & = & E_{\phi }\left(b_{t},p_{t}\right) \end{array}$$

(5) $$\begin{array}{ccc} \tilde{b_{t}} & = & D_{\theta }\left(c_{t},p_{t}\right) \end{array}$$

The decoder generates the approximate k-space navigator signals $\tilde{b_{t}}$ at time *t*, given the 2D latent vectors $c_{t}$$=\left[\begin{array}{cc} c_{\mathrm{card},t} & c_{\mathrm{resp},t} \end{array}\right]$ and the conditional vector $p_{t}$. The decoder is implemented using fully connected layers, whose parameters are denoted by θ. We use a progressive architecture, starting with a dense layer, and the spatial dimension of the features are grown gradually. We concatenate the latent vectors and the conditional vector and feed them as inputs to the decoder. The latent vectors $c_{t}$ follow the conditional distribution $p(c_{t}|b_{t})$, which is approximated by $q_{\phi }\left(c_{t}|b_{t}\right)$. Similar to the classical VAE setting, we model $q_{\phi }\left(c_{t}|b_{t}\right)$$=N(z_{t},\Sigma_{t})$ as a Gaussian distribution, where the mean vector $z_{t}$$=\left[\begin{array}{cc} z_{\mathrm{card},t} & z_{\mathrm{resp},t} \end{array}\right]$ and the diagonal covariance matrix $\Sigma_{t}$$=\mathrm{diag}(\sigma_{t})$, where σ$=\left[\begin{array}{cc} \sigma_{\mathrm{card},t} & \sigma_{\mathrm{resp},t} \end{array}\right]$, are derived by the encoder network $E_{\phi }$. The coefficients $c_{t}$ can be obtained by sampling from $q_{\phi }\left(c_{t}|b_{t}\right)$.

The standard VAE cost function is specified by $C\left(\theta ,\phi \right)=\sum_{t=1}^{N_{\mathrm{frames}}}E_{t}\left(\theta ,\phi \right)$, where $E_{t}$ is given by

(6) $$\begin{array}{ccc} E_{t}\left(\theta ,\phi \right) & = & \underset{\mathrm{data}\;\mathrm{term}}{\underset{︸}{-\frac{1}{2\sigma^{2}}E_{c_{t}∼q_{\phi }\left(b_{i}\right)}\left[∥D_{\theta }\left(c_{t},p_{t}\right)-b_{t}{∥}^{2}\right]}}\\ & & \;-\;\underset{\mathrm{prior}}{\underset{︸}{KL[q_{\phi }\left(c_{t}\right)||p\left(c_{t}\right)]}}. \end{array}$$

The first term is the data term that compares the output of the decoder to the k-space navigator signals. The second term is the Kullback–Leibler (KL) divergence of the distribution $q_{\phi }\left(c_{i}\right)$ from the desired prior distribution $p(c_{i})$. The KL divergence term encourages the latent distribution $q_{\phi }\left(c_{t}|b_{t}\right)$ to follow a zero mean and unit variance Gaussian distribution, which ensures that the latent vectors will be uncorrelated. In this work, we assume $p\left(c_{i}\right)=N\left(0,I\right)$, where I is the identity matrix. In this case, the KL divergence can be explicitly evaluated as

$$L\left(c_{t}\right)=\frac{-\log\left[\det\left(\Sigma_{t}\right)\right]-n+\mathrm{trace}\left(\Sigma_{t}\right)+z_{t}^{T}z_{t}}{2}$$

where we assume a latent space of dimension *n*.

In our experience, the use of the latent vectors estimated by the above VAE model with G-SToRM can provide good image recovery. However, the latent vectors are often not disentangled and hence may not be interpretable. In particular, each latent signal may capture a mix of cardiac and respiratory motion components. In this work, we are interested in disentangling the impact of cardiac and respiratory components, which will facilitate the generation of images with specific cardiac or respiratory phases. Several disentanglement priors were introduced in machine learning, including beta-VAE [25], to improve the interpretability of the latent vectors. These approaches rely on information theoretic or statistical priors, which are dependent on the distribution of the latent vectors.

Motivated by self-gating methods that use band-pass filtering with preset bands [26,27] to separate the phase signals, we use a disentanglement strategy based on the frequency range of the latent vectors. In this work, we constrain the cardiac and respiratory mean vectors to be sparse linear combinations of real exponentials with frequencies between 0.1–0.6 Hz and 0.8–2 Hz, respectively. In particular, we constrain mean vectors $z_{\mathrm{card},t}$ and $z_{\mathrm{resp},t}$ to be

(7) $$\begin{array}{ccc} z_{\mathrm{card},t} & = & Ce \end{array}$$

(8) $$\begin{array}{ccc} z_{\mathrm{resp},t} & = & Rd \end{array}$$

where C and R are matrices whose rows are sinusoidal basis functions with frequencies in respective bands, and e and d are the corresponding coefficients. We note that the matrices C and R are not necessarily orthogonal matrices because of the delays used for magnetization recovery when no data are acquired.

We note that the estimation of the latent vectors is challenging in the $T_{1}$ mapping context because the k-space navigator signals are modulated by inversion pulses, as seen in Figure 2A. In particular, the strength of the navigator signals are very low when the myocardium and blood are nulled. We note that prior approaches relied on several pre-processing steps on the k-space navigators, including rejecting unreliable sections and interpolation [17]. To minimize the modulation of the latent vectors, we penalize the $\ell_{1}$ norm of the coefficients e and d to encourage them to be sparse. We pose the VAE with the bandlimited disentanglement strategy as

$$\begin{array}{ccc} \left\{\theta^{*},\phi^{*}\right\} & = & \arg\min_{\theta ,\phi }C\left(\theta ,\phi \right)+\lambda \left({∥e∥}_{\ell_{1}}+\lambda_{2}{∥d∥}_{\ell_{1}}\right),\\ & & \;e={\left(C^{T}C\right)}^{-1}C^{T}\;z_{\mathrm{card},t}\\ & & \;d={\left(R^{T}R\right)}^{-1}R^{T}\;z_{\mathrm{resp},t}. \end{array}$$

We use gradient descent to minimize the above cost function.

### 3.3. Image Reconstruction

Once the VAE optimization is completed, the mean vectors $z_{t}$ in (4) are indicative of the cardiac and respiratory phases. This information, together with the inversion time, may be used to bin the data to respective phases. Since each of the bins is often heavily undersampled, the standard practice is to use a constrained reconstruction algorithm such as MRI multi-tasking [16] to perform the recovery. However, multi-tasking requires dedicated k-space navigators to estimate the subspace factors. We instead rely on the implicit structural bias of CNNs to constrain the reconstructions, motivated by deep image prior [28] and its extensions to dynamic MRI [18]. In particular, CNNs can learn natural images faster than noise, which can be used as a prior to constrain imaging-based inverse problems [28]. The extension of this work to dynamic MRI [18] seeks to combine the information from multiple cardiac/respiratory phases, thus further improving the reconstructions.

We model the 2D image frames in the time series as the output of a CNN generator:

(9) $$x_{t}=G_{\zeta }\left(z_{t},p_{t}\right),\;i=1,\cdots ,M$$

in response to the concatenation of the latent vectors and the parameters, as shown in Figure 2B. A progressive CNN architecture similar to [18] is used to realize $G_{\zeta }$, which facilitates the learning of the information from multiple time points. In particular, the filters at the top layers of the CNN capture the fine image details that can be learned from multiple time instances. By contrast, the coarse features at the earlier layers are specific to the shape of the objects and contrasts, which may vary with time.

The cost function for the scheme can be described as follows:

(10) $$C\left(\zeta \right)=\underset{\mathrm{data}\;\mathrm{term}}{\underset{︸}{\sum_{t=1}^{N_{\mathrm{frames}}}{∥A_{t}\left(G_{\zeta }\left(z_{t},p_{t}\right)\right)-b_{t}∥}^{2}}}+\eta \underset{\mathrm{net}\;\mathrm{reg}.}{\underset{︸}{∥J_{c}G_{\zeta }{\left(z\right)∥}^{2}}}.$$

Here, z${}_{t}$ are the latent vectors estimated using the auto-encoder as described in Section 3.2, while $p_{t}$ is the inversion signal. Both variables are held fixed during the image reconstruction step. Image recovery is thus reformulated as the learning of the CNN generator parameters ζ from the entire set of measured k-t space measurements. Here, $A_{t}$ corresponds to the multi-channel non-uniform Fourier transform operators that vary from time frame to time frame. We add the network regularization term to make the learning of the generator more stable. Here, $J_{c}G$ denotes the Jacobian of the mapping, which restricts the capacity of the model. The illustration of the reconstruction scheme is shown in Figure 2B. The main distinctions of the proposed approach with [18] is the pre-learning of the latent vectors rather than the joint learning of both ζ and z. The main benefits of this two-step approach are the quite significant reduction in computation time and the ability to work with larger datasets. To further speed up the convergence, we propose a stochastic training strategy. Specifically, we randomly divide the number of all frames into different batches at each iteration, followed by stochastic gradient descent. These improvements enable us to extend the approach to the $T_{1}$ mapping setting.

**Figure 3:**
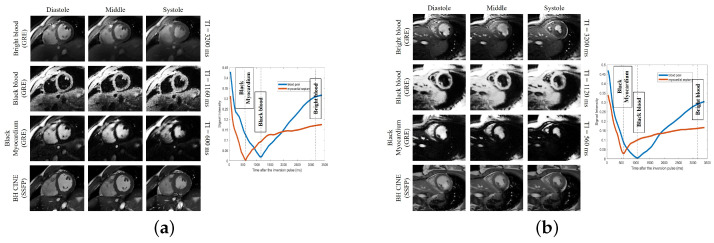
(**a**) Results on subject #1. (**b**) Results on subject #6. Generation of synthetic breath-hold cine data with different contrasts. We show the results on two subjects in (**a**,**b**), respectively. In each figure, the top three image rows on the left correspond with the synthetic breath-hold cine at three representative inversion times. The images from the 2D conventional gated Cartesian cine images acquired using the balanced SSFP sequence are also shown in the bottom row for comparison purposes. The right side of each sub-figure is the plot of the signal evolutions of the myocardium and blood pool.

Once the generator parameters ζ are learned, one can generate images with arbitrary cardiac/respiratory phases and inversion time using (9), as shown in Figure 3. These images can be used to evaluate cardiac function and to quantify the $T_{1}$ parameters, described in the section below.

### 3.4. $T_{1}$ Mapping

We propose two approaches to estimate the $T_{1}$ maps, which we term the retrospective binning and generative approaches, respectively. Both approaches rely on fingerprint matching, where we use Bloch simulations to generate the fingerprints for different $T_{1}$ values as in [29]. Since we allow a recovery time after each inversion block, we expect a reduced dependency of estimated $T_{1}$ maps on the flip angle. Since we directly account for the impact of the excitation pulses and the repetition time, the fingerprint matching approach is expected to be more accurate than the analytical exponential models.

#### 3.4.1. Generative Approach

Once the learning is complete, we fix the latent vectors z as the desired cardiac and respiratory phase (e.g., diastole and peak inspiration) and vary the inversion signal *p* with the desired step-size as shown in Figure 2C. The generator is excited with the constant latent signals and varying inversion signals to generate images with different inversion times. The $T_{1}$ maps are derived by comparing the fingerprints to the pre-computed dictionary. Here, we use the entire Bloch dictionary for the matching; the combinations of inversion times and the phases, which are not seen during imaging, are interpolated by the generative model. This approach considerably simplifies the evaluation of the $T_{1}$ maps. However, we note that the images generated by this approach are not necessarily data-consistent, since the specific combinations of phases may not have happened during the imaging process. We hence compare the $T_{1}$ maps derived using this approach with the retrospective binning strategy, which is data-consistent.

#### 3.4.2. Retrospective Binning

Once the learning is complete, the generator can be fed with the latent vectors and inversion signal at any arbitrary time point to obtain the corresponding image. In the retrospective binning strategy, we identify the time-instants when the latent vectors are in a specified cardiac and respiratory phase as illustrated in Figure 2D. We feed the generator the corresponding latent vectors and inversion signal to generate the corresponding images. Note that the loss function in (10) involves these images, and hence they are more data-consistent than the generative approach in Figure 2C. In the retrospective setting, we use a sub-dictionary from the Bloch dictionary, consisting of the above time-instants. The $T_{1}$ maps are obtained by matching the images with the sub-dictionary.

## 4. Experiments

All the data used in this work are acquired on a 3T MR750W scanner (GE Healthcare, Waukesha, WI, USA).

### 4.1. Implementation Details

We note that the proposed scheme in this work is totally unsupervised and hence no fully sampled training data are needed. The parameters in the dense auto-encoder as well as the parameters of the CNN are learned from the undersampled k-t space data of the specific subject being worked on, resulting in a subject-specific representation. In other words, we solve the networks based on each different subject.

#### 4.1.1. Dense Auto-Encoder

The encoder $E_{\phi }$ and decoder $D_{\theta }$ used for motion estimation are implemented using dense multilayer perceptron, and ReLU activation is used. The encoder $E_{\phi }$ has four layers, and the four layers have 25, 50, 100, and 70 features, respectively. The decoder $D_{\theta }$ also has four layers, and the four layers have 70, 100, 50, and 25 features, respectively.

We determine the parameter λ using trial and error on one of the subjects such that the Fourier transform of the latent vectors has a well-defined peak. Lower values of λ often result in latent vectors with residual modulation corresponding to inversion preparations. Once the parameter is determined on one of the datasets, they are fixed for other datasets.

#### 4.1.2. CNN Image Generator

We use a CNN image generator with three inputs (2D latent vectors and a 1D conditioning vector involving the inversion time). The CNN generates a two-channel output image, which corresponds to the real and imaginary parts in the MR images. Eight layers are used to implement the generator, and the total number of trainable parameters in the generator is about 15 times the image size of one image frame. For the convolutional layers, leaky ReLU activation is used for the generator except for the last layer, where tanh is used as the activation function. Random initialization is used to initialize all the networks. Note that we progressively increase the dimensions of the features, starting with small dimensions.

The parameter η is determined by using trial and error on one of the subjects. Specifically, we tune the parameter η on one of the subjects to have the best visual image quality. Then, we keep η the same for all datasets.

### 4.2. Phantom Used for the Validation of $T_{1}$ Maps

We use a commercially available (Caliber MR, Boulder, CO, USA) Essential System Phantom containing NIST traceable human tissue mimic solutions measured with landmark accuracy and precision to determine the accuracy of the $T_{1}$ maps. The phantom is built with 14 NiCl_2_ samples, 14 MnCl_2_ samples, 14 proton density samples, and 1 CuSO_4_ fiducial sample. In this study, we image the 14 NiCl_2_ samples with different known $T_{1}$ values.

We use the proposed inversion recovery sequence with three different settings to determine the dependence of the $T_{1}$ maps on the flip angle and the delay time used for recovery. In setting I, we use flip angle $\alpha =3^{\circ }$ with a delay time of 500 ms. In setting II, we set flip angle $\alpha =14^{\circ }$ with a delay time of 500 ms. In setting III, we use flip angle $\alpha =14^{\circ }$ with a delay time of 5000 ms. The conventional 2D peripheral pulse gating (PPG)-triggered MOLLI [5(3)3] data [6] are also acquired for comparison purposes. Except for the sequence parameters shown in Figure 1C, some other imaging parameters are set as follows for the proposed inversion recovery sequence: slice thickness = 8 mm, TR = 8 ms. Sequence parameters for MOLLI are: TR/TE =2.55/1.056 ms, flip angle $=35^{\circ }$, readout bandwidth =868 Hz.

### 4.3. Acquisition Scheme and Pre-Processing for In Vivo Data

An in vivo study is performed using the short-axis orientation without contrast. Considering that flip angle $3^{\circ }$ will give us a poor contrast between myocardium and blood pool, we use setting II in the phantom study for the in vivo data acquisition. In particular, we use the flip angle $\alpha =14^{\circ }$ and delay time =500 ms for the data acquisition in a free-breathing and ungated fashion. The TR for the acquisition is 8 ms, and the acquisition time for one slice is 34 s. All the datasets are acquired using the AIR coil developed by GE HealthCare (Waukesha, WI, USA). For comparison purposes, we also acquire the breath-hold cine using the 2D SSFP sequence and the 2D conventional MOLLI images for $T_{1}$ mapping estimation. The parameters for the breath-hold SSFP sequence are: TR/TE = 3.48/1.52 ms, flip angle $=49^{\circ }$ and readout bandwidth =488 Hz. Six subjects (four healthy volunteers and two patients; 21 to 51 years old; four females) are involved in this study. The public information for the six subjects is summarized in Table 1. The Institutional Review Board at the University of Iowa approved the acquisition of the data, and written consents were obtained from all subjects.

We use an algorithm developed in-house to pre-select the coils that provide the best signal-to-noise ratio in the region of interest. We then estimate the coil sensitivity maps using ESPIRiT [30]. A total of 4000 spirals are acquired for each slice. During the reconstructions, we bin every five spirals corresponding to 40 ms temporal resolution for each frame in the time series. We omit the data from the first inversion time to eliminate the impact of transients during the reconstruction.

## 5. Results

### 5.1. Estimation of the Latent Vectors

**Figure 4:**
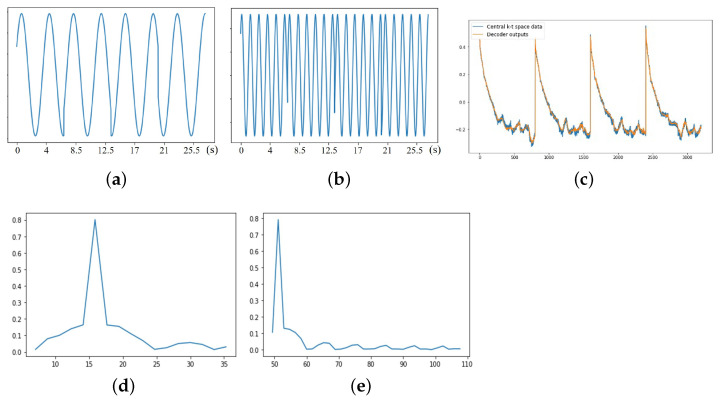
(**a**) Respiratory latent vector. (**b**) Cardiac latent vector. (**c**) VAE training comparison. (**d**) Respiratory rate (bpm). (**e**) Heart rate (bpm). Estimated latent vectors from subject #2, who has bradycardia. (**a**,**b**) are the respiratory and cardiac latent vectors obtained from the encoder. The *x*-axis is the time in seconds. (**c**) shows the comparison between the output of the decoder and the central k-t space data from one of the coils. (**d**,**e**) correspond to the Fourier transform of the respiratory and cardiac latent vectors. The bandlimited constraint, together with the sparsity penalty, enables us to recover the cardiac and respiratory signals reliably, even in the presence of strong signal modulations. From (**e**), we see that the estimated heart rate from the dense VAE is 53 bpm, which is roughly the same as the average heart rate of 55 bpm, which was obtained on the scanner.

In Figure 4, we show the estimated latent vectors using the dense auto-encoder. We note from Figure 4c that the VAE output closely matches the k-t space navigators, which shows the modulation resulting from the inversion pulses. The estimated cardiac and respiratory signals are shown in Figure 4a,b, while their Fourier coefficients are shown in Figure 4d,e. We note that the $\ell_{1}$ norm minimization resulted in relatively sparse Fourier coefficients. The abrupt jumps in the cardiac latent vector result from the delays added for magnetization recovery, when no data are acquired.

### 5.2. Retrospective Binning

**Figure 5:**
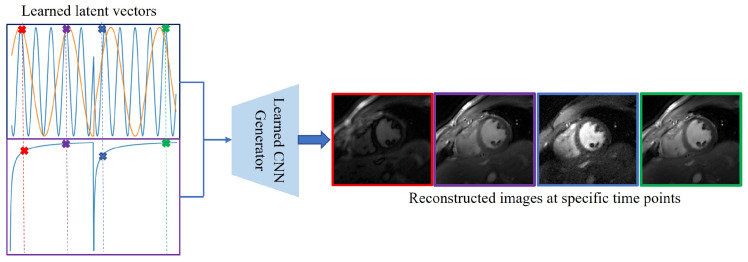
Illustration of learned CNN generator and latent vectors. The 2D latent vectors are learned from the dense-auto-encoder, and we also have the 1D conditioning vector involving the inversion time. The 3D latent vectors are then fed into the trained CNN generator, and the generator then generates the desired image frames in the time-series. We showed four generated images in this figure. The latent vectors corresponding to these four images are also marked in the figure.

In Figure 5, we show the retrospectively binned images from one of the subjects. Here, we pick frames with similar cardiac and respiratory phases, but with different inversion times and hence different contrasts. These images are directly involved in the loss function specified by (10) and hence are data-consistent. Similarly, one can identify cardiac phases closer to a specific inversion time to obtain cine-like movies, which can be used for the estimation of quantitative parameters. However, it is often challenging to find segments with matching respiratory phases and contrasts with the retrospective binning strategy. By contrast, the generation of the synthetic images discussed below is easier.

### 5.3. Generation of Synthetic Images

The proposed scheme learns a generative model from the measured k-t space data. Hence, one can excite the generator with arbitrary combinations of latent vectors and inversion signals to generate synthetic images on demand. We now illustrate two important modes.

#### 5.3.1. Synthetic Breath-Hold Cine Data with Different Contrasts

We fix the inversion signal and respiratory phase, and only vary the cardiac phase to generate synthetic breath-held cine images. The inversion times may also be varied to generate cine data with different contrasts as shown in Figure 3. In Figure 3, three representative $T_{1}$-weighted breath-hold cine images are shown for two subjects. We showed the bright blood cine, black blood cine, and black myocardium cine obtained from the proposed scheme. For better visualization purposes, we chose a small region of interest, which contains the heart, to show in the image. Additionally, we scaled the color map to better display the images. The corresponding time points for the generation of the three $T_{1}$-weighted breath-hold cine images are visible as dashed lines in the signal evolution curves, also shown in Figure 3. From Figure 3, we see that both the black blood and bright blood cine images resolve the contrast between myocardium and blood pool well.

#### 5.3.2. Images with Different Inversion Times

**Figure 6:**
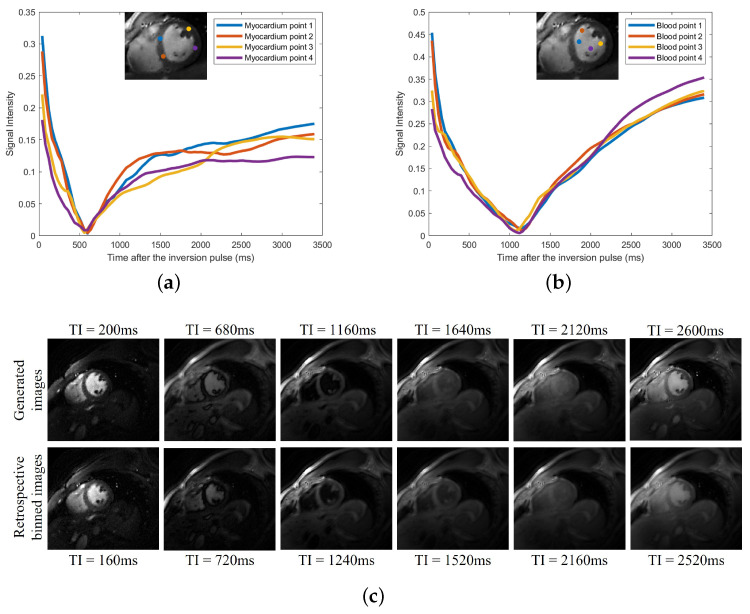
(**a**) Signal evolutions of myocardium. (**b**) Signal evolutions of blood. (**c**) Signal evolutions of blood. Generation of synthetic images with different inversion times. We fix the latent phase signals to the desired phases: diastole at end inspiration. We only vary the inversion signal and then generate the images in the time-series. We then plot the signal evolutions based on the generated image frames. In (**a**,**b**), we show the plots of the signal evolutions of the myocardium and blood pool at four different positions, respectively. In the top row of (**c**), six generated image frames at six different inversion times are shown. In the bottom row of (**c**), we show six similar images we picked from the original reconstructed (i.e., feed the learned phase latent vectors and inversion time signal into the learned CNN generator) time-series, and the corresponding inversion time is also shown at the bottom of each image.

Similarly, the latent phase signals can be fixed to desired phases (e.g., diastole at end inspiration), and the inversion signal can be varied to generate images with arbitrary inversion times. We illustrate this in Figure 6. We note that during the short acquisition, each phase may not experience all possible inversion times. The SToRM learns to interpolate the signal from the available measurements.

### 5.4. Accuracy of the $T_{1}$ Maps

**Figure 7:**
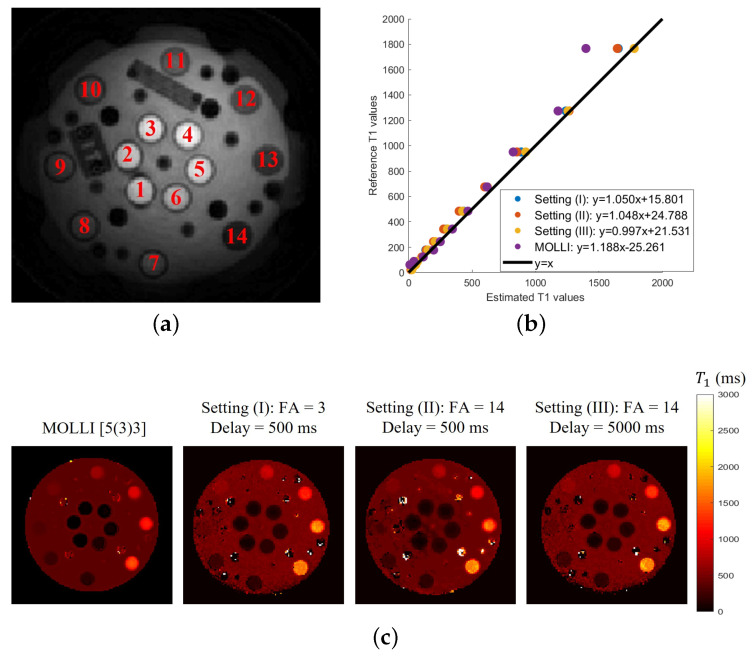
(**a**) Image of 14 NiCl_2_ samples. (**b**) Comparison of the $T_{1}$ values. (**c**) $T_{1}$ mappings for different cases. Results of the phantom study. (**a**) shows the 14 NiCl_2_ samples used in the phantom study. The $T_{1}$ mappings from MOLLI and three different settings based on the proposed inversion recovery sequence are shown in (**b**). The comparison between the mean $T_{1}$ values in each of the 14 NiCl_2_ regions for different settings and the reference $T_{1}$ values of these 14 NiCl_2_ samples is shown in (**c**).

Results of the phantom study are shown in Figure 7 and Table 2. In Figure 7c, the $T_{1}$ mappings were generated for the three settings and compared to the 2D MOLLI result. In Figure 7b, we computed the mean $T_{1}$ values in each of the 14 NiCl_2_ regions for different settings and compared them to the reference $T_{1}$ values of these 14 NiCl_2_ samples.

Our experiments also show that all of the estimates are more reliable than the MOLLI results. In particular, the MOLLI scheme will significantly underestimate the values for $T_{1}$ values greater than 1200 ms. The correlation analyses between the estimated $T_{1}$ values and the reference $T_{1}$ values for different settings are shown in Table 2. The R-squared value [31] and the intraclass correlation coefficient (ICC) [32] metrics are used for the correlation analysis. For ICC, we computed ICC(A,1), which gives an estimate of the reliability of the method if an absolute agreement between different measurements is desired. From the quantitative results, we see that the results obtained from the proposed inversion recovery scheme show a strong positive correlation relationship ($R^{2}>0.99$ and ICC(A,1) > 0.99) with the reference results, as well as slightly better estimation than the MOLLI scheme. We note that the 2D MOLLI is unable to measure small $T_{1}$ values; it failed to estimate the $T_{1}$ values of the first three NiCl_2_ samples (with reference $T_{1}$ values 21.94 ms∼43.79 ms). We note that the most consistent results over the entire range of $T_{1}$ values are yielded by setting III, which corresponds to the five-second delay time. Settings I and II, which correspond to FA = 3${}^{\circ }$ and FA = 14${}^{\circ }$, show a mild underestimation at the high $T_{1}$ values, which are significantly smaller than that exhibited by MOLLI. Both of these settings are comparable, indicating that the flip angle has minimal impact on the $T_{1}$ estimates.

### 5.5. $T_{1}$ Estimation from Free-Breathing and Ungated MRI

**Figure 8:**
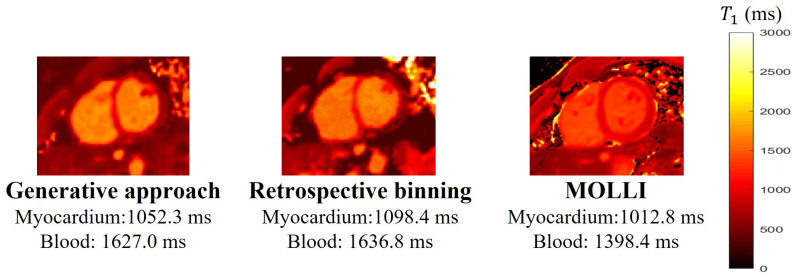
Comparisons of the $T_{1}$ mappings obtained from both retrospective binning and the generative approach, and the 2D MOLLI [5(3)3] based on subject #1. From this, we can see that retrospective binning and the generative approach can provide comparable results.

This section shows the results of the $T_{1}$ maps estimated using the proposed scheme. Specifically, we trained the generators for each subject. We use the generative approach described in Section 3.4.1 to estimate the images, where we fixed the cardiac phase as the diastole phase. The $T_{1}$ mappings obtained from the proposed generative approach and retrospective binning are compared to the 2D MOLLI results, based on one subject, in Figure 8. From the comparison, we see that retrospective binning and the generative approach yield comparable results. Because the generative approach is simpler to use, we used this scheme for the remaining experiments.

The average $T_{1}$ values for myocardium, left blood pool, and right blood pool estimated from MOLLI and the proposed scheme are shown in Table 3. Over all six subjects, the average $T_{1}$ values for the myocardium between the proposed scheme and MOLLI have no significant difference (p=0.7844). However, for the $T_{1}$ value of the blood, the estimation from the proposed scheme is consistently higher than the estimation from MOLLI. This mismatch is explained by the phantom study, where MOLLI underestimated the $T_{1}$ values when the $T_{1}$ values are around 1500 ms∼2000 ms. Based on the existing studies [33,34], the native $T_{1}$ value of the blood on the 3T scanner is around 1500 ms∼1800 ms. We suppose that one of the reasons for the underestimation of large $T_{1}$ values with MOLLI in this study comes from imperfect gating. In this work, peripheral pulse gating is used for the 2D MOLLI sequence.

### 5.6. Cardiac Function Analysis

**Figure 9:**
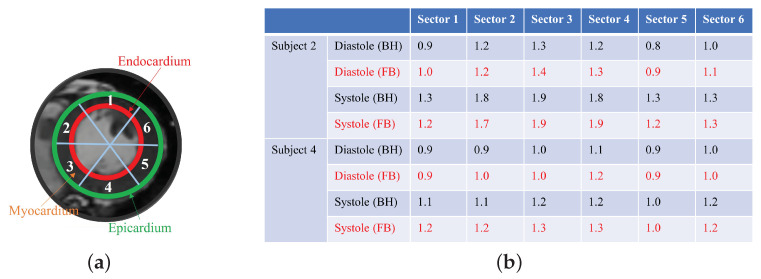
(**a**) The six sectors of the myocardium. (**b**) Comparison of the area of each sectors from FB and BH (cm^2^). Left ventricle wall analysis. We compared the areas of the six sectors of the myocardium obtained from the generated cine and the breath-hold cine. From the quantitative results in the table, we can see that the generated cine using the proposed scheme is able to provide results similar to those of the breath-hold cine.

We generated synthetic breath-hold cine data using the proposed scheme, which are then used to estimate the cardiac function analysis. We compare the left ventricle (LV) wall analysis results with the 2D conventional gated Cartesian cine images acquired using the 2D conventional gated Cartesian balanced SSFP cine sequence in Figure 9. We divided the myocardium into six sectors and calculated the area of each sector for both the diastole phase and the systole phase, and we then compared the areas obtained from both the generated breath-hold cine and the 2D conventional gated Cartesian cine. The LV wall analysis was performed using the commercial software Segment (Medviso). We assume that the border of the endocardium and the epicardium account for 20 percent of the LV wall. From the quantitative results, we can see that the results from the generated breath-hold cine are consistent with the results obtained from the 2D conventional gated Cartesian cine.

## 6. Discussion and Conclusions

In this study, we proposed a manifold-based recovery scheme for the joint recovery of inversion recovery prepared free-breathing and ungated cardiac MRI. We represent each image in the time-series as a non-linear function of three variables: the cardiac and respiratory phases and inversion time. The non-linear function is realized using a CNN generator, while the CNN parameters and the phase information are estimated from the measured k-t space data. The data were acquired using a spiral GRE sequence with intermittent inversion pulses. We use a two-step strategy to realize a computationally efficient algorithm. In particular, we use a dense conditional auto-encoder to estimate the cardiac and respiratory phases from the central multi-channel k-space samples acquired at each frame. The latent vectors of the auto-encoder are constrained to be bandlimited functions with appropriate frequency bands, which enables the disentanglement of the latent vectors into cardiac and respiratory phases even when the data are acquired with intermittent inversion pulses. Once the phases are estimated, we pose the image recovery as the learning of the parameters of the CNN generator from the measured k-t space data. The learned CNN generator is used to generate synthetic data on demand by feeding it with appropriate latent vectors. The framework enables the generation of cine movies with different inversion contrasts as well as the estimation of the $T_{1}$ maps with specific phases.

Phantom studies demonstrate that the proposed scheme is able to provide $T_{1}$ estimation with good accuracy and precision, comparable to MOLLI. The phantom experiments show that the proposed $T_{1}$ estimates are not sensitive to the specific choice of flip angles. The experiments also show that the proposed scheme is less biased than MOLLI at higher $T_{1}$ values. We note that the results from the proposed scheme are slightly biased when the delay used for magnetization recovery is short. We attribute this slight bias to transient effects; even though we omitted the data from the first inversion block, there may be residual transient effects in the first several inversion blocks, especially when the delay time for magnetization recovery is small. By contrast, the longer delays will ensure that the transient effects are minimal. In the future, we will explore magnetization preparation strategies to further minimize the slight bias. The in vivo studies on six subjects also show good agreements between the cardiac $T_{1}$ mapping estimation from the proposed scheme and MOLLI [33,34]. We note that there is an inconsistency between the $T_{1}$ values of blood between MOLLI and the proposed scheme. This discrepancy can be explained by the higher bias of MOLLI, especially in the range 1500 ms∼2000 ms.

The proposed framework also offers the ability to measure cardiac function using a synthetic breath-hold cine. The experimental results show that the synthetic cine is able to provide comparable cardiac function analysis results compared to the results from the 2D conventional gated Cartesian cine. A benefit with the proposed scheme is its ability to generate multiple contrasts, some of which offer improved visualization of myocardial borders and may facilitate improved analysis.

The proposed reconstruction algorithm is general enough to be applied to other sequences, including the free-running transient balanced SSFP acquisition with variable flip angles and inversion pulses as in [15], as well as the sequence proposed in [17]; we will consider such extensions in the future, on a larger patient cohort, to compare the proposed method with the methods proposed in [15,17]. We also plan to study the clinical feasibility of the proposed methods on a larger patient cohort. In this work, we used the spoiled gradient echo (SPGR) with the spiral trajectories for the data acquisition on a 3T scanner. A benefit of this sequence is its robustness to banding artifacts, compared to balanced SSFP, especially at high field strengths. The longer spiral readouts provide an improved inflow contrast between the myocardium and the blood pool. We did not observe significant blurring from B_0_ inhomogeneities and gradient imperfections in this study.

## Figures and Tables

**Table 1:** Information for the six subjects involved in this study.

	Gender	Age	Health Condition
Subject 1	M	21	Low LVEF
Subject 2	F	24	Low heart rate
Subject 3	F	27	Healthy
Subject 4	M	51	Healthy
Subject 5	F	49	Healthy
Subject 6	F	29	Healthy

**Table 2:** The correlation analysis between the estimated $T_{1}$ values and the reference $T_{1}$ values for different settings for phantom study.

	Setting (I)	Setting (II)	Setting (III)	MOLLI
$R^{2}$	0.999	0.997	0.998	0.986
ICC(A,1)	0.996	0.994	0.998	0.971

**Table 3:** The average $T_{1}$ values for myocardium, left blood pool, and right blood pool estimated from MOLLI and the proposed scheme. Results from six subjects are shown in the table.

Subject No.	Methods	Myocardium	Left Blood Pool	Right Blood Pool
Subject 1	MOLLI	1012.8	1369.6	1427.2
	Proposed	1052.3	1643.2	1610.8
Subject 2	MOLLI	1103.9	1547.3	1455.0
	Proposed	1009.9	1638.6	1684.7
Subject 3	MOLLI	1069.2	1498.2	1509.3
	Proposed	1063.3	1717.3	1714.3
Subject 4	MOLLI	1080.1	1447.4	1443.6
	Proposed	1104.1	1718.5	1679.0
Subject 5	MOLLI	1049.7	1423.8	1447.6
	Proposed	1089.2	1617.8	1618.9
Subject 6	MOLLI	1048.8	1573.6	1535.3
	Proposed	1082.4	1770.7	1789.0

## Data Availability

The data presented in this study are available on request from the corresponding author. The data have not been made publicly available due to the confidentiality of the data.
